# Compromised Dynamic Cerebral Autoregulation in Patients with Epilepsy

**DOI:** 10.1155/2018/6958476

**Published:** 2018-02-07

**Authors:** Shan Lv, Zhen-Ni Guo, Hang Jin, Xin Sun, Meiyan Jia, Hongyin Ma, Yudan Lv, Quanli Qiu, Jia Liu, Yi Yang

**Affiliations:** ^1^Department of Neurology, The First Hospital of Jilin University, Changchun, China; ^2^Clinical Trial and Research Center for Stroke, Department of Neurology, The First Hospital of Jilin University, Changchun, China; ^3^Shenzhen Institutes of Advanced Technology, Chinese Academy of Sciences, Xueyuan Avenue, Shenzhen University Town, Shenzhen, China

## Abstract

**Objective:**

The aim of this study is to analyze dynamic cerebral autoregulation (dCA) in patients with epilepsy.

**Methods:**

One hundred patients with epilepsy and 100 age- and sex-matched healthy controls were recruited. Noninvasive continuous cerebral blood flow velocity of the bilateral middle artery and arterial blood pressure were recorded. Transfer function analyses were used to analyze the autoregulatory parameters (phase difference and gain).

**Results:**

The overall phase difference of patients with epilepsy was significantly lower than that of the healthy control group (*p* = 0.046). Furthermore, patients with interictal slow wave had significant lower phase difference than the slow-wave-free patients (*p* = 0.012). There was no difference in overall phase between focal discharges and multifocal discharges in patients with epilepsy. Simultaneously, there was no difference in mean phase between the affected and unaffected hemispheres in patients with unilateral discharges. In particular, interictal slow wave was an independent factor that influenced phase difference in patients with epilepsy (*p* = 0.016).

**Conclusions:**

Our study documented that dCA is impaired in patients with epilepsy, especially in those with interictal slow wave. The impairment of dCA occurs irrespective of the discharge location and type. Interictal slow wave is an independent factor to predict impaired dCA in patients with epilepsy.

**Clinical Trial Identifier:**

This trial is registered with NCT02775682.

## 1. Introduction

The relationship between epileptic seizures and stroke is intimate but complex. Epilepsy not only is a common neurologic sequela of stroke, but also could sometimes herald a stroke [1, 2]. Since the first epilepsy preceding stroke was described in early 1982 [3], postepilepsy stroke had been noted. Simultaneously, several studies reported that patients with epilepsy tend to have a higher stroke risk [4–6]. The potential mechanisms remain unclear. Studies have reported both supranormal demands of cerebral blood flow and disruption of neurovascular coupling after epileptic discharge [7, 8]. These abnormalities in cerebral hemodynamics could well be an important mechanism in the development of postepilepsy stroke.

Dynamic cerebral autoregulation, a mechanism to maintain cerebral blood flow, is a reliable method to evaluate cerebrovascular function and has been proven to be critical for the occurrence [9], development, and prognosis [10] of ischemic stroke. Because seizure has potential effect on cerebral hemodynamics, cerebral autoregulation may be of particular importance to patients with epilepsy to maintain stable cerebral blood flow. It has been demonstrated in animals that cerebral autoregulation is disrupted during both seizures and the subsequent postictal state, and impaired cerebral autoregulation may be involved in the pathogenesis of ischemic brain lesions [11, 12]. However, few studies with limited numbers have examined cerebral autoregulation in humans with epilepsy to date.

In the study, we hypothesize that cerebral autoregulation is impaired in patients with epilepsy during the interictal state, which has a role in the occurrence of postepilepsy stroke. We use transfer function method, the most commonly used method to quantify dynamic cerebral autoregulation [13] based on spontaneous fluctuations of blood pressure and cerebral blood flow velocity, to identify our hypothesis. If this assumption is valid, cerebral autoregulation may become a potential intervention target for preventing postepilepsy stroke.

## 2. Materials and Methods

The prospective study design was approved by the ethics committee of the First Hospital of Jilin University under the guidelines of the Declaration of Helsinki (1964). All the participants/guardians signed the written informed consent forms. The study is listed at clinicaltrials.gov/under identifier NCT02775682.

### 2.1. Participants

Patients with epilepsy who were already scheduled for EEG examination were recruited from the Department of Neurology, First Hospital of Jilin University, between April 2016 and May 2017. Each patient received a diagnosis of epilepsy by two experienced neurological physicians separately according to the operational clinical definition of epilepsy recommended by the International League Against Epilepsy (ILAE) in 2013 [14]. All patients underwent CT/MRI examination when first diagnosed with epilepsy. We placed no restriction on age and sex. The exclusion criteria included (1) patients with status epilepticus; (2) intracranial and/or extracranial major vascular stenosis/occlusion diagnosed by a transcranial Doppler (EMS-9PB, Delica, China) and carotid ultrasound (IU22, Phillips, Andover, MA), based on the criteria defined by Wong et al. [15]; (3) a prior symptomatic cerebral vascular disease; (4) a history of brain trauma, brain tumor, encephalitis, and other symptomatic neurological diseases; (5) a history of arterial hypertension, cardiovascular disease, diabetes, hyperlipidemia, current arrhythmia, hyperthyroidism, and anemia, which may undermine hemodynamic stability, or inability to cooperate sufficiently to complete the cerebral autoregulation examination; (6) insufficient bilateral temporal bone windows for insonation of the middle cerebral artery; and (7) intolerance to cerebral autoregulation measurements. Age- and sex-matched healthy controls without epilepsy were recruited from the same region who otherwise met the same eligibility criteria as the patients.

### 2.2. Dynamic Cerebral Autoregulation Measurement

The procedures of dynamic cerebral autoregulation assessment were performed on the basis of the white paper published in 2016 by the International Cerebral Autoregulation Research Network [16]. Continuous cerebral blood flow velocity was recorded noninvasively using transcranial cerebral Doppler device (MultiDop X2, DWL, Sipplingen, Germany) in bilateral middle cerebral artery at a depth of 45 to 60 mm. Spontaneous arterial blood pressure was recorded using a servo-controlled plethysmograph (Finometer PRO, Netherlands) on the subject's middle finger of the right hand positioned at heart level. The analog output of arterial blood pressure was plugged into the transcranial cerebral Doppler device where two channels of cerebral blood flow velocity (bilateral) and the signal of arterial blood pressure were recorded simultaneously. In order to confirm stability of respiration, end-tidal CO_2_ was monitored using a capnograph with a facemask attached to the nasal cannula.

In the group of patients with epilepsy, dynamic cerebral autoregulation measurement was performed after EEG examination was completed. All the participants were accessed during 8 to 11 am to minimize the diurnal variation of dynamic cerebral autoregulation. The participants were told to avoid alcohol intake and exercise for at least 12 hours. Caffeinated drinks and the ingestion of a heavy meal were also abstained from for a minimum of 4 hours. The measurement was performed in a quiet, dedicated research laboratory with minimized visual or acoustic stimulation, at a controlled temperature of 22 to 24°C. First, the subjects were told to breathe normally in a supine position for 15 min to measure baseline arterial blood pressure (Omron 711) and heart rates. Then, both cerebral blood flow velocity and arterial blood pressure were recorded simultaneously for 10 min. All the measurements were performed by one experienced operator.

### 2.3. Data Analysis

Recorded data were processed using MATLAB software (Math Works, Natick, MA, USA). The raw waveforms were sampled at 100 Hz for both arterial blood pressure and cerebral blood flow velocity. Alignment of the raw waveforms was achieved using a cross-correlation function to remove possible time lags caused between the devices. Mean values of the signals within each cardiac cycle were calculated and interpolated by third-order polynomial spline to achieve beat-to-beat signals with a uniform sampling rate at 10 Hz. A third-order Butterworth low-pass filter (cutoff at 0.5 Hz) was then applied as an antialias filter before downsampling the data to 1 Hz. Dynamic cerebral autoregulation was evaluated using transfer function analysis [16, 17]. Fast Fourier transform was used to transform time series of blood pressure and cerebral blood flow velocity to the frequency domain. The transfer function between arterial blood pressure and cerebral blood flow velocity was calculated as the quotient of the cross-spectrum of the two signals and the autospectrum of arterial blood pressure in the low frequency domain (0.06–0.12 Hz) to obtain frequency-dependent estimates of phase difference and gain, where the derived parameters are considered most relevant to autoregulation hemodynamics [18]. At the same time, coherence was calculated to estimate the reliability of the relationship between the two signals at the frequency domain, and the later statistical analysis was performed only if coherence of the parameters was >0.5 [19].

### 2.4. Statistical Analysis

Continuous variables with a normal distribution, including phase difference and gain, were expressed as mean (standard deviation), while variables with skewed distribution were expressed as median (interquartile range). Discrete variables were expressed as absolute values and percentages. The intergroup differences were tested using the *t*-test. We defined the overall phase difference/gain as mean phase difference/gain of bilateral cerebral hemispheres. Univariate and multivariate linear regression were used to assess the association of dynamic cerebral autoregulation parameters and clinical parameters including sex, age, discharge types (focal discharges and multifocal discharges), duration (years), discharge period (discharge at waking versus at sleep state), interictal slow wave, and antiepileptic drugs therapy. All the data were analyzed using the Statistical Program for Social Sciences version 23.0 (SPSS, IBM, West Grove, PA, USA). *p* < 0.05 was considered statistically significant.

## 3. Results

### 3.1. Participant Characteristics

**Figure 1:**
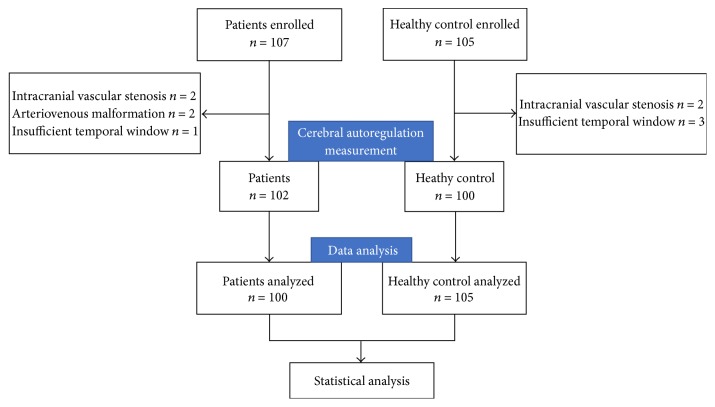
Participant enrollment.

Of 107 patients with epilepsy and 105 age- and sex-matched healthy controls enrolled in this study, 100 patients with epilepsy and 100 healthy controls completed the cerebral autoregulation measurement (Figure 1). Among the patients with epilepsy, 44 (44%) patients had focal discharges (discharge originated from a single site) and 56 (56%) patients had multifocal discharges (discharge originated from more than one lobe or within same lobe of bilateral cerebral but appeared nonsynchronously). The media seizure duration was 6.6 (1,10) years. Fourteen subjects were with new-onset epilepsy. Sixteen patients had epileptic discharges combined with interictal slow wave, and 38 (38%) patients had only epileptic discharges during sleep. Among 66 patients with temporal region seizures, 31 (47%) patients had unilateral discharges, and 35 (53%) patients had bilateral discharges. No patients had a clinical seizure onset during the dynamic cerebral autoregulation measurement. Neurologic examinations were normal in all participants. We did not find any significant differences in sex, age, mean blood pressure, heart rate, or end-tidal carbon dioxide between patients with epilepsy and those in the control group. The clinical characteristics of participants are shown in Table 1.

### 3.2. Autoregulatory Parameters between Patients with Epilepsy and the Control Group

**Figure 2:**
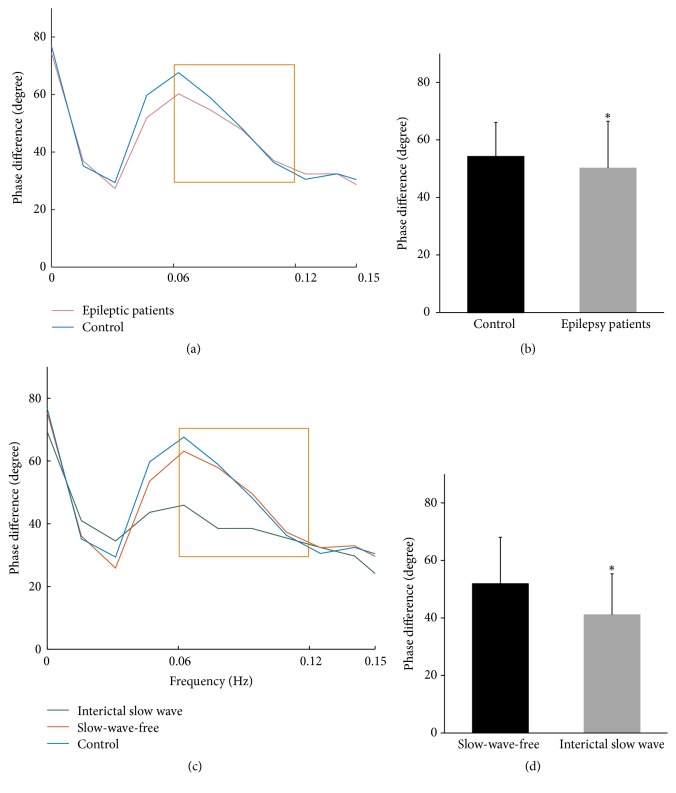
*The autoregulatory parameter and statistical distributions in overall epileptic patients and epileptic patients with/without slow wave*. (a) The autoregulatory parameter (phase difference) derived from the transfer function in overall epileptic patients. (b) Statistical distributions of the phase difference in overall epileptic patients. (c) The phase difference derived from the transfer function in epileptic patients with/without slow wave. (d) Statistical distributions of the phase difference in epileptic patients with/without slow wave. In (a) and (c), frames in orange represent specific frequency domain (0.06–0.12 Hz). In (b) and (d), bars denote means; whiskers denote standard error. *N* = 16 for interictal slow wave patients; *n* = 84 for slow-wave-free patients. *∗* indicates statistically different (*p* < 0.05).

The overall phase difference of patients with epilepsy was significantly lower than that of the control group (50.20 ± 16.28 versus 54.23 ± 11.84 degree, 95% confidence interval [CI] −8.01 to −0.07, *p* = 0.046), as shown in Figures 2(a) and 2(b). There were no significant differences between the overall gain of the two groups (0.87 ± 0.30 versus 0.84 ± 0.25 cm/s/mmHg, 95% CI −0.05 to 0.11, *p* = 0.415).

Interestingly, patients with interictal slow wave (*n* = 16) had significant lower phase difference than slow-wave-free patients (*n* = 84) (41.11 ± 14.23 versus 51.93 ± 16.14 degree, 95% CI −19.03 to 2.60, *p* = 0.012; Figures 2(c) and 2(d)). The overall gain of these two groups had no significant differences (0.92 ± 0.34 versus 0.86 ± 0.29 cm/s/mmHg, 95% CI −0.13 to 0.24, *p* = 0.534).

### 3.3. Autoregulatory Parameters of Different Groups of Patients

**Figure 3:**
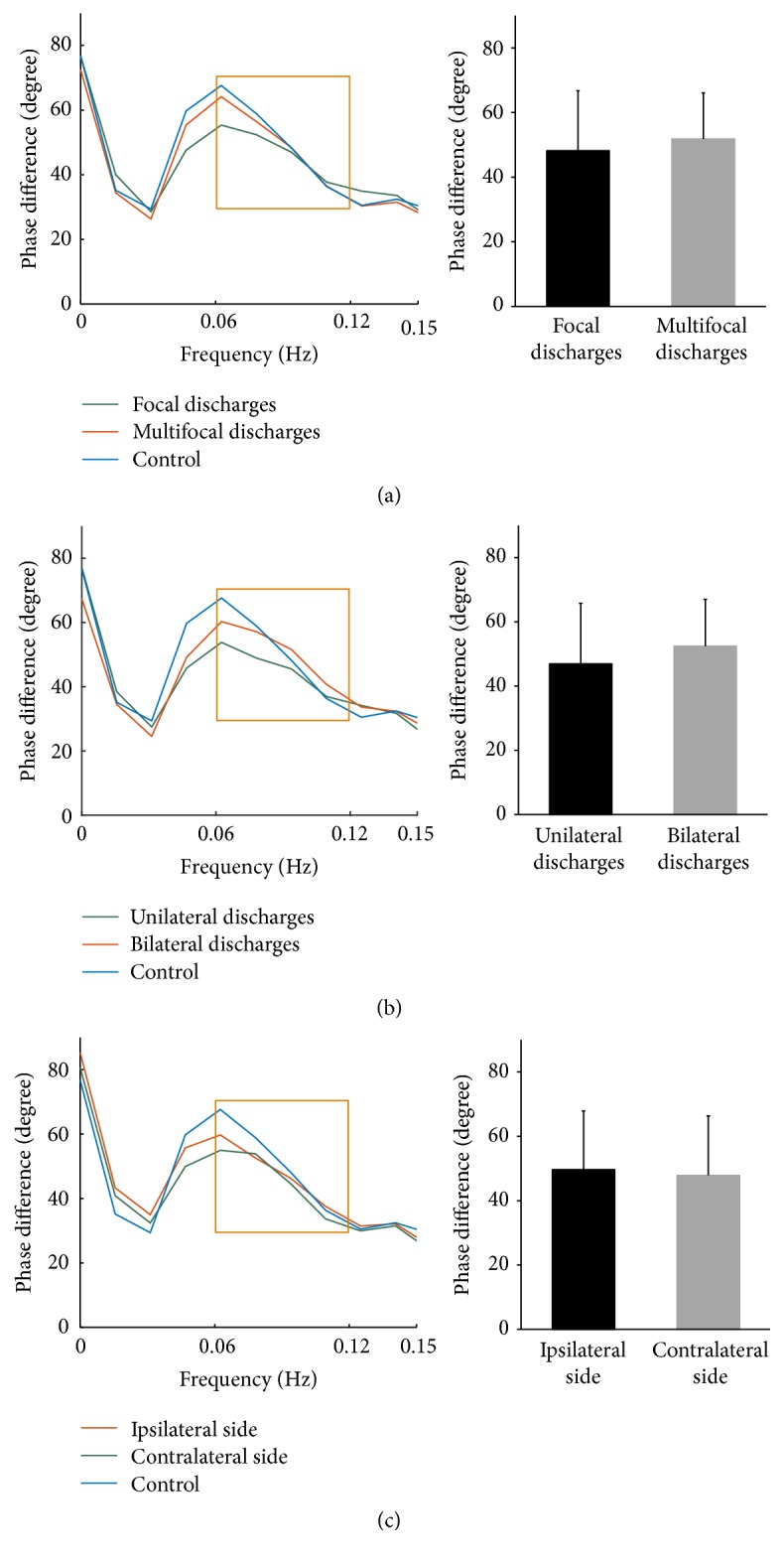
*The autoregulatory parameter and statistical distributions in each group*. (a) Phase difference derived from the transfer function (left) and its statistical distributions (right) in patients with focal discharges and multifocal discharges. (b) Among patients with temporal seizure, the overall phase difference (left) and its statistical distributions (right) in patients with unilateral discharges and bilateral discharges. (c) Mean phase difference (left) and its statistical distributions (right) of ipsilateral side and contralateral side in patients with unilateral discharges. Denotations by lines and frames are similar to those in Figure 2. *N* = 44 for patients with focal discharges; *n* = 56 for patients with multifocal discharges; among 66 patients with temporal seizure, *n* = 31 for unilateral discharges and *n* = 35 for bilateral discharges; *n* = 36 for patients with unilateral discharges.

No significant differences of the dynamic cerebral autoregulation parameters were noted between patients with focal discharges (*n* = 44) and patients with multifocal discharges (*n* = 56) (phase difference 48.16 ± 18.52 versus 51.80 ± 14.25 degree, 95% CI −10.36 to 3.10, *p* = 0.286; gain 0.86 ± 0.30 versus 0.88 ± 0.30 cm/s/mmHg, 95% CI −0.14 to 0.10, *p* = 0.757; Figure 3(a)).

Among patients with temporal region seizure, no significant differences of the overall phase and gain between unilateral discharges (*n* = 31) and bilateral discharges (*n* = 35) were observed (phase difference 48.98 ± 18.10 versus 50.36 ± 16.42 degree, 95% CI −7.11 to 9.86, *p* = 0.749; gain 0.90 ± 0.28 versus 0.87 ± 0.38 cm/s/mmHg, 95% CI −0.19 to 0.14, *p* = 0.753; Figure 3(b)). To identify whether epileptic discharges could influence cerebral autoregulation in the contralateral hemisphere, we analyzed 36 patients with unilateral discharges and did not find significant differences of the two autoregulatory parameters between ipsilateral side and contralateral side (phase difference 49.62 ± 18.21 versus 47.88 ± 18.43 degree, 95% CI −4.63 to 8.10, *p* = 0.584; gain 0.89 ± 0.27 versus 0.88 ± 0.31 cm/s/mmHg, 95% CI −0.09 to 0.09, *p* = 0.995; Figure 3(c)).

### 3.4. Univariable and Multivariable Analyses

The clinical parameters used in the univariable and multivariable analysis are shown in Table 2. In the univariable model, interictal slow wave was related to lower phase difference (*p* = 0.016). The multivariable model included gender, age, and interictal slow wave. Interictal slow wave was an independent factor that influenced phase difference. No factors were detected associated with gain after multivariable analysis.

## 4. Discussion

In this study, we found the autoregulatory parameter, phase difference, was impaired in patients with epilepsy. There were no differences of the impairment between focal discharges and multifocal discharges and no differences between affected and unaffected hemispheres. In particular, interictal slow wave was an independent factor that influenced phase difference values in patients with epilepsy. These findings may increase understanding of the underlying mechanism where patients with epilepsy tend to have higher risk of stroke and provide a potential intervention target to prevent postepilepsy stroke.

The first case of epilepsy preceding stroke was described in early 1982 by Barolin [3]. Several years later, a case-control study by Shinton and colleagues showed that preexisting epilepsy was more common in the stroke group, which suggested that epilepsy could sometimes herald a stroke [1]. Subsequently, studies with larger amount of patients supported Shinton's hypothesis in both elderly and young patients [4]. Recently, a prospective study by Sillanpää et al. with five decades of follow-up showed that patients with childhood-onset epilepsy had higher MRI abnormalities, including those with epilepsy in remission, which may be a predictor of clinically evident stroke [20]. The potential mechanisms underlying this phenomenon remain unclear. Except for the use of antiepileptic drugs and the lifestyles of patients with epilepsy that have been demonstrated to be risk factors for stroke (such as smoking, physical inactivity, and certain unhealthy diet choice), epileptic seizures were thought to be an essential cause [21]. Shinton et al. detected that, among the patients who had partial motor epilepsy, a hemiplegia developed on the same side as the epileptic focus in most cases [1]. It was probably the earliest evidence of this kind. Olesen et al. reported a higher risk of stroke in patients with untreated epilepsy [22]. Another study showed the risk of stroke was higher in patients with epilepsy without any vascular risk factors (such as hypertension, atrial fibrillation, cardiovascular disease, diabetes, or hyperlipidemia) [5]. In this study, none of the participants had any identified vascular risk factors, and we did not find any significant influences of clinical factors on dynamic cerebral autoregulation parameters containing smoking and antiepileptic drugs therapy. Because of the altered hemodynamics and hypoperfusion during both ictal and interictal events [7], we speculated that disrupted cerebral hemodynamics caused by epileptic discharges participated in the subsequent stroke.

It is widely accepted that during epileptic seizures, the energy metabolism of the cerebrum increases accompanied by the rising neuronal activity, leading to an increase in cerebral blood flow simultaneously. Studies accessed by fMRI and SPECT have confirmed this [23, 24]. However, several studies have shown the opposite cerebral blood flow changes [25, 26]. For example, frontal region hypoperfusion has been documented in patients with temporal seizures [27, 28]. As the intrinsic mechanism to maintain cerebral perfusion, cerebral autoregulation dilates arterioles to increase cerebral blood flow in the ictal and postictal state [7]. Cerebral blood flow changes underlie an exhaustion of cerebral autoregulation capability [25]. Several decades ago Hascoet et al. demonstrated in animals that autoregulation of cerebral blood flow was impaired during both seizures and the subsequent postictal state [12]. They thought this persistent impaired cerebral autoregulation was relevant in the pathogenesis of hemorrhagic or ischemic brain lesions. However, in Hascoet et al.'s study, newborn piglets were studied at 20 to 90 min after cessation of seizures, and the correlations between cerebral blood flow and mean arterial pressure in this period represented cerebral autoregulation in the postictal state. Cerebral autoregulation during subclinical onset and interictal period has not been described yet. Our study focuses on the interictal period, and compromised cerebral autoregulation during this period suggested that it was not only clinical seizure onset but also interictal epileptic discharges that influenced cerebral hemodynamics. Patients with lower cerebral autoregulation may be prone to hypoperfusion during epileptic events. In addition, we did not find significant differences in dynamic cerebral autoregulation parameters between patients with focal discharges and multifocal discharges, nor did we find the ipsilateral side and contralateral side in patients with unilateral discharges. That is, the cerebral autoregulation of epilepsy patients was impaired bilaterally despite the discharge location and type, suggesting the possibility of distant changes induced by chronic epileptic discharges. Analogously, a study by Dütsch and colleagues demonstrated that temporal lobe epilepsy surgery improved the dynamic cerebral autoregulation parameters gain and phase bilaterally, regardless of the side of surgery. They thought a decrease in interictal epileptic activity mostly led to a decreased sympathetic cerebrovascular modulation after the surgery and thus improved cerebral autoregulation capability [29].

The potential mechanisms of impaired cerebral autoregulation in the interictal period are unclear. Since none of our patients had clinical seizure onset before and during measurement, we believe that repetitive interictal epileptic discharges as well as their underlying etiology influenced cerebrovascular function through a series of neuroendocrine mechanisms, thus affecting cerebral autoregulation. Our hypothesis has some evidence. Above all, despite the controversy, cerebral hemodynamic alterations were detected during variable epileptic discharges [23, 27, 28]. Further, epileptic seizures have been proven to disrupt the neurovascular coupling [7], which is another distinct mechanism to regulate cerebral blood flow. This suggested that epileptic seizures not only cause cerebral hemodynamics alteration but also influence cerebrovascular function. The research of Gómez-Gonzalo et al. showed that astrocyte activation resulted from Ca^2+^ elevation and participated in the control of neurovascular coupling and vasomotor response during epileptic activity. However, they observed that compared with the ictal discharges the efficacy of interictal discharges was too poor to elicit cerebral arteriole response, which differed from our findings [30]. In our opinion, as Ca^2+^ elevation and isolated astrocytes activation were indeed seen during interictal discharges, it was likely that chronic and repetitive interictal discharges elicited neurovascular coupling alteration and cerebrovascular dysfunction combined with occasional ictal events. Moreover, Rosengarten et al. observed that neurovascular coupling might have identical mechanisms with cerebral autoregulation [31], which helped explain our results. In addition, interictal autonomic nervous system dysfunction was seen in patients with epilepsy [32, 33], which affected the cerebral autoregulation [34, 35]. Autonomic dysfunction was thought to accelerate with duration and depends on the degree of seizure control [36]. Similarly, Dütsch et al. considered that enhanced autonomic cerebrovascular function could explain the cerebral autoregulation recovery after surgery in patients with temporal lobe epilepsy [29].

Another important finding of this study was that slow wave was an independent factor that influenced phase difference in patients with epilepsy. Slow wave is a fundamental cortical rhythm that generally emerges in deep nonrapid eye movement sleep [37], mainly composed of delta slowing. In the waking state, slow wave is produced by lesions in both cortical gray matter and subcortical white matter [38, 39] and is generally thought to represent structural or metabolic dysfunction [40]. The pathogenesis of interictal slow wave remains poorly understood. Keller and colleagues found an increase in neuronal firing during discharge as well as a diminished rate of neuronal activity during the slow wave, corresponding to a period of relative inhibition, suggesting that slow wave represented inhibition of brain function. Additionally, physiologic events that resulted in the interictal discharge were not limited to the seizure focus [41]. There are also perspectives that interictal regional delta slowing represents the epileptogenic process, for its locations are striking in accordance with the epileptic focus [40]. Furthermore, several studies demonstrated a strong relationship between interictal slow wave and diminished rate of neuronal activity in the relevant regions [41, 42]. In this study, we recognized that cerebral autoregulation of patients with interictal slow wave was impaired even and interictal slow wave was an independent predictor of dynamic cerebral autoregulation capability. We speculate that interictal slow wave plays an essential role in the impairment of dynamic cerebral autoregulation through unknown mechanisms and may have an association with postepilepsy stroke. Epidemiologic studies with large sample are needed to confirm this hypothesis.

The study has some limitations. First, the dynamic cerebral autoregulation and EEG were examined separately. Thus, we cannot identify whether the patients had subclinical epileptic discharges during the dynamic cerebral autoregulation measurement, which may influence the cerebral hemodynamics. Second, epilepsy was caused by different reasons, such as juvenile myoclonic epilepsy or mesial temporal lobe sclerosis, which may result in difficulty in explaining the mechanisms of cerebral autoregulation impairment. However, our limitation is not to distinguish the different causes of epilepsy. Furthermore, dementia, a potential factor to influence cerebral autoregulation, is common in patients with epilepsy [43]. Not including the cognitive function test is a limitation of our study.

Our findings raised new questions. It is not easy to determine whether disruption of dynamic cerebral autoregulation during the chronic epileptic phase is just a consequence of epileptic discharges or that it can also contribute to discharges. Further work is required to explore this issue. The cause-and-effect relationship between interictal slow wave and dynamic cerebral autoregulation should also be explored further.

## 5. Conclusions

The present study documented that dynamic cerebral autoregulation capability is impaired in the patients with epilepsy, especially in those with interictal slow wave. Cerebral autoregulation disruption occurs irresponsive of the discharge location and type, suggesting hemodynamic changes exceeding the epileptogenic focus. Interictal slow wave is an independent factor to predict impaired cerebral autoregulation in patients with epilepsy.

## Figures and Tables

**Table 1:** Baseline characteristics of patients with epilepsy and the control group.

	Patients (*n* = 100)	Control group (*n* = 100)	*p*
Age (year)	32.7 ± 11.8	32.1 ± 10.3	0.703
Gender, male/female	40/60	40/60	1
Smoking, *n* (%)	17 (17.0%)	10 (10.0%)	0.092
Mean blood pressure (mmHg)	87.3 ± 8.5	86.4 ± 7.5	0.399
Mean MCA velocity	67.69 ± 12.34	64.82 ± 12.41	0.102
Heart rate (beats/min)	70.7 ± 8.1	70.4 ± 7.6	0.773
End-tidal CO_2_ (mmHg)	37.6 ± 1.7	37.1 ± 1.8	0.527
Discharge types			
Focal discharges, *n* (%)	44 (44)		
Multifocal discharges, *n* (%)	56 (56)		
Epileptic discharge sites			
Temporal region, *n* (%)	66 (66)		
Frontal region, *n* (%)	13 (13)		
Multiple regions, *n* (%)	21 (21)		
Discharge state			
Wake, *n* (%)	62 (62)		
Sleep, *n* (%)	38 (38)		
Interictal EEG discharge wave			
Sharp waves, *n* (%)	40 (40)		
Sharp-wave complex, *n* (%)	73 (73)		
Spikes, *n* (%)	12 (12)		
Spike-wave complex, *n* (%)	20 (20)		
Slow waves, *n* (%)	16 (16)		
AED therapy, *n* (%)	48 (48)		

MCA, middle cerebral artery; AED, antiepileptic drugs.

**Table 2:** Univariable and multivariable analysis of clinical parameters associated with autoregulatory parameters.

	Univariable analysis				Multivariable analysis			
	Phase difference		Gain		Phase difference		Gain	
	*β*	*p*	*β*	*p*	*β*	*p*	*β*	*p*
Gender	0.004	0.971	0.037	0.713	0.042	0.681	0.078	0.491
Age	−0.030	0.768	−0.137	0.175	−0.010	0.917	−0.196	0.065
Smoking	−0.108	0.291	−0.101	0.324				
Epileptic discharge sites	−0.056	0.624	−0.054	0.596				
Interictal slow wave	−0.241	0.016^ab^	0.069	0.496	−0.249	0.016^bc^		
Duration	0.167	0.108	−0.154	0.139				
Epileptic discharge type	0.101	0.318	0.034	0.736				
Discharge state	0.006	0.953	−0.011	0.914				
AED therapy	−0.017	0.875	0.127	0.231				

^a^Nominally significant values (*p* < 0.1) included in the multivariable model; ^b^*p* value < 0.05 (statistically different); ^c^independent factor that influences cerebral autoregulation.

## References

[1] Shinton R. A., Zezulka A. V., Gill J. S., Beevers D. G. (1987). The frequency of epilepsy preceding stroke. Case-control study in 230 patients. *The Lancet*.

[2] Silverman I. E., Restrepo L., Mathews G. C. (2002). Poststroke seizures. *JAMA Neurology*.

[3] Barolin G. S. (1982). The cerebrovascular epilepsies. *Electroencephalography and Clinical Neurophysiology*.

[4] Cleary P., Shorvon S., Tallis R. (2004). Late-onset seizures as a predictor of subsequent stroke. *The Lancet*.

[5] Téllez-Zenteno J. F., Matijevic S., Wiebe S. (2005). Somatic comorbidity of epilepsy in the general population in Canada. *Epilepsia*.

[6] Brigo F., Tezzon F., Nardone R. (2014). Late-onset seizures and risk of subsequent stroke: a systematic review. *Epilepsy & Behavior*.

[7] Schwartz T. H. (2007). Neurovascular coupling and epilepsy: hemodynamic markers for localizing and predicting seizure onset. *Epilepsy Currents*.

[8] Harris S., Boorman L., Bruyns-Haylett M. (2014). Contralateral dissociation between neural activity and cerebral blood volume during recurrent acute focal neocortical seizures. *Epilepsia*.

[9] Reinhard M., Gerds T. A., Grabiak D. (2008). Cerebral dysautoregulation and the risk of ischemic events in occlusive carotid artery disease. *Journal of Neurology*.

[10] Reinhard M., Rutsch S., Lambeck J. (2012). Dynamic cerebral autoregulation associates with infarct size and outcome after ischemic stroke. *Acta Neurologica Scandinavica*.

[11] Clozel M., Daval J. L., Monin P., Dubruc C., Morselli P. L., Vert P. (1985). Regional cerebral blood flow during bicuculline-induced seizures in the newborn piglet: effect of phenobarbital. *Developmental Pharmacology and Therapeutics*.

[12] Hascoet J. M., Monin P., Vert P. (1988). Persistence of impaired autoregulation of cerebral blood flow in the postictal period in piglets. *Epilepsia*.

[13] Meel-van den Abeelen A. S. S., van Beek A. H. E. A., Slump C. H., Panerai R. B., Claassen J. A. H. R. (2014). Transfer function analysis for the assessment of cerebral autoregulation using spontaneous oscillations in blood pressure and cerebral blood flow. *Medical Engineering & Physics*.

[14] Fisher R. S., Acevedo C., Arzimanoglou A. (2014). ILAE official report: a practical clinical definition of epilepsy. *Epilepsia*.

[15] Wong K. S., Li H., Chan Y. L. (2000). Use of transcranial doppler ultrasound to predict outcome in patients with intracranial large-artery occlusive disease. *Stroke*.

[16] Claassen J. A., Meel-Van Den Abeelen A. S., Simpson D. M. (2015). Transfer function analysis of dynamic cerebral autoregulation: A white paper from the International Cerebral Autoregulation Research Network. *Journal of Cerebral Blood Flow & Metabolism*.

[17] Giller C. A. (1990). The frequency-dependent behavior of cerebral autoregulation. *Neurosurgery*.

[18] van Beek A. H. E. A., Claassen J. A. H. R., Rikkert M. G. M. O., Jansen R. W. M. M. (2008). Cerebral autoregulation: an overview of current concepts and methodology with special focus on the elderly. *Journal of Cerebral Blood Flow & Metabolism*.

[19] Kuo T. B., Chern C., Sheng W., Wong W., Hu H. (2016). Frequency domain analysis of cerebral blood flow velocity and its correlation with arterial blood pressure. *Journal of Cerebral Blood Flow & Metabolism*.

[20] Sillanpää M., Anttinen A., Rinne J. O. (2015). Childhood-onset epilepsy five decades later. A prospective population-based cohort study. *Epilepsia*.

[21] Jin J., Chen R., Xiao Z. (2016). Post-epilepsy stroke: a review. *Expert Review of Neurotherapeutics*.

[22] Olesen J. B., Abildstrøm S. Z., Erdal J. (2011). Effects of epilepsy and selected antiepileptic drugs on risk of myocardial infarction, stroke, and death in patients with or without previous stroke: a nationwide cohort study. *Pharmacoepidemiology and Drug Safety*.

[23] Zhao M., Suh M., Ma H., Perry C., Geneslaw A., Schwartz T. H. (2007). Focal increases in perfusion and decreases in hemoglobin oxygenation precede seizure onset in spontaneous human epilepsy. *Epilepsia*.

[24] Andersen A. R., Waldemar G., Dam M. (1990). SPECT in the presurgical evaluation of patients with temporal lobe epilepsy—a preliminary report. *Neurosurgical Aspects of Epilepsy*.

[25] Szirmai I., Molnar M., Czopf J., Borsics K. (1977). Spreading epileptiform discharges and cortical regional blood flow in rabbits. *Electroencephalography and Clinical Neurophysiology*.

[26] Zhao M., Nguyen J., Ma H., Nishimura N., Schaffer C. B., Schwartz T. H. (2011). Preictal and ictal neurovascular and metabolic coupling surrounding a seizure focus. *The Journal of Neuroscience*.

[27] van Paesschen W., Dupont P., van Driel G., van Billoen H., Maes A. (2003). SPECT perfusion changes during complex partial seizures in patients with hippocampal sclerosis. *Brain*.

[28] Rabinowicz A. L., Salas E., Beserra F., Leiguarda R. C., Vazquez S. E. (1997). Changes in regional cerebral blood flow beyond the temporal lobe in unilateral temporal lobe epilepsy. *Epilepsia*.

[29] Dütsch M., Devinsky O., Doyle W., Marthol H., Hilz M. J. (2004). Cerebral autoregulation improves in epilepsy patients after temporal lobe surgery. *Journal of Neurology*.

[30] Gómez-Gonzalo M., Losi G., Brondi M. (2011). Ictal but not interictal epileptic discharges activate astrocyte endfeet and elicit cerebral arteriole responses. *Frontiers in Cellular Neuroscience*.

[31] Rosengarten B., Huwendiek O., Kaps M. (2001). Neurovascular coupling and cerebral autoregulation can be described in terms of a control system. *Ultrasound in Medicine & Biology*.

[32] Druschky A., Hilz M. J., Hopp P. (2001). Interictal cardiac autonomic dysfunction in temporal lobe epilepsy demonstrated by [^123^I]metaiodobenzylguanidine-SPECT. *Brain*.

[33] Diehl B., Diehl R. R., Stodieck S. R. G., Ringelstein E. B. (1997). Spontaneous oscillations in cerebral blood flow velocities in middle cerebral arteries in control subjects and patients with epilepsy. *Stroke*.

[34] Blaber A. P., Bondar R. L., Stein F. (1997). Transfer function analysis of cerebral autoregulation dynamics in autonomic failure patients. *Stroke*.

[35] Castro P. M., Santos R., Freitas J., Panerai R. B., Azevedo E. (2014). Autonomic dysfunction affects dynamic cerebral autoregulation during Valsalva maneuver: comparison between healthy and autonomic dysfunction subjects. *Journal of Applied Physiology*.

[36] Persson H., Ericson M., Tomson T. (2007). Heart rate variability in patients with untreated epilepsy. *Seizure*.

[37] Csercsa R., Dombovári B., Fabó D. (2010). Laminar analysis of slow wave activity in humans. *Brain*.

[38] Pellegrino G., Tombini M., Curcio G. (2017). Slow activity in focal epilepsy during sleep and wakefulness. *Clinical EEG and Neuroscience*.

[39] Gloor P., Ball G., Schaul N. (1977). Brain lesions that produce delta waves in the EEG. *Neurology*.

[40] Gambardella A., Gotman J., Cendes F., Andermann F. (1995). Focal intermittent delta activity in patients with mesiotemporal atrophy: a reliable marker of the epileptogenic focus. *Epilepsia*.

[41] Keller C. J., Truccolo W., Gale J. T. (2010). Heterogeneous neuronal firing patterns during interictal epileptiform discharges in the human cortex. *Brain*.

[42] Altafullah I., Halgren E., Stapleton J. M., Crandall P. H. (1986). Interictal spike-wave complexes in the human medial temporal lobe: typical topography and comparisons with cognitive potentials. *Electroencephalography and Clinical Neurophysiology*.

[43] Toth P., Tarantini S., Csiszar A., Ungvari Z. (2017). Functional vascular contributions to cognitive impairment and dementia: mechanisms and consequences of cerebral autoregulatory dysfunction, endothelial impairment, and neurovascular uncoupling in aging. *American Journal of Physiology-Heart and Circulatory Physiology*.

